# Supplementary material for: Preclinical Evidence of STAT3 Inhibitor Pacritinib Overcoming Temozolomide Resistance via Downregulating miR-21-Enriched Exosomes from M2 Glioblastoma-Associated Macrophages
Source: J Clin Med. 2019 Jul 2;8(7):959. doi: 10.3390/jcm8070959 (PMC6678764; doi:10.3390/jcm8070959)
Supplement: Supplementary file 1 [file jcm-08-00959-s001.pdf]

SUPPLEMENTARY INFORMATION

Preclinical evidence of STAT3 inhibitor, Pacritinib, overcomes temozolomide resistance via down-regulating miR-21-enriched exosomes from M2 glioblastoma-associated macrophages

Authors:

Hao-Yu Chuang [1,2,3], Yu-kai Su [4,5,6,7], Heng-Wei Liu[4,5,6,7], Chao-Hsuan Chen[8,9,10,11], Shao-Chih Chiu [8,9,10,11], Der-Yang Cho [8,9,10,11], Shinn-Zong Lin[12,13], Yueh-Sheng Chen[14*], Chien-Min Lin [4,5,6,7*]

Affiliation:

1. Graduate Institute of Clinical Medical Science, China Medical University, Taichung, Taiwan
2. Department of Neurosurgery, An Nan Hospital, China Medical University, Tainan, Taiwan
3. Department of Neurosurgery, China Medical University Beigang Hospital, Yunlin, Taiwan
4. Graduate Institute of Clinical Medicine, College of Medicine, Taipei Medical University, Taipei City 11031, Taiwan
5. Department of Neurology, School of Medicine, College of Medicine, Taipei Medical University, Taipei City 11031, Taiwan
6. Division of Neurosurgery, Department of Surgery, Taipei Medical University-Shuang Ho Hospital, New Taipei City 23561, Taiwan
7. Taipei Neuroscience Institute, Taipei Medical University, Taipei 11031, Taiwan
8. Center for Cell Therapy, China Medical University Hospital, Taichung, Taiwan
9. Drug Development Center, China Medical University, Taichung, Taiwan
10. Graduate Institute of Biomedical Sciences, China Medical University, Taichung, Taiwan
11. Department of Neurosurgery, China Medical University Hospital, Taichung, Taiwan
12. Bioinnovation Center, Buddhist Tzu Chi Medical Foundation, Hualien, Taiwan
13. Department of Neurosurgery, Tzu Chi University, Hualien Tzu Chi Hospital, Buddhist Tzu Chi Medical Foundation, Hualien, Taiwan

14. Department of Biomedical Imaging and Radiological Science, China Medical University, Taichung, Taiwan

***Corresponding authors:**

Yueh-Sheng Chen, PhD.: Department of Biomedical Imaging and Radiological Science, China Medical University, Taichung, Taiwan; Tel.: +886-2-2490088 ext. 8881; Fax: 886-2-2248-0900; E-mail: yuehsc@mail.cmu.edu.tw

Chien-Min Lin, MD., PhD, Department of Neurosurgery, Taipei Medical University - Shuang Ho Hospital, New Taipei City 23561, Taiwan, Tel: +886-2-2490088 ext. 8885; Fax: +886-2-2248-0900; E-mail addresses: m513092004@tmu.edu.tw

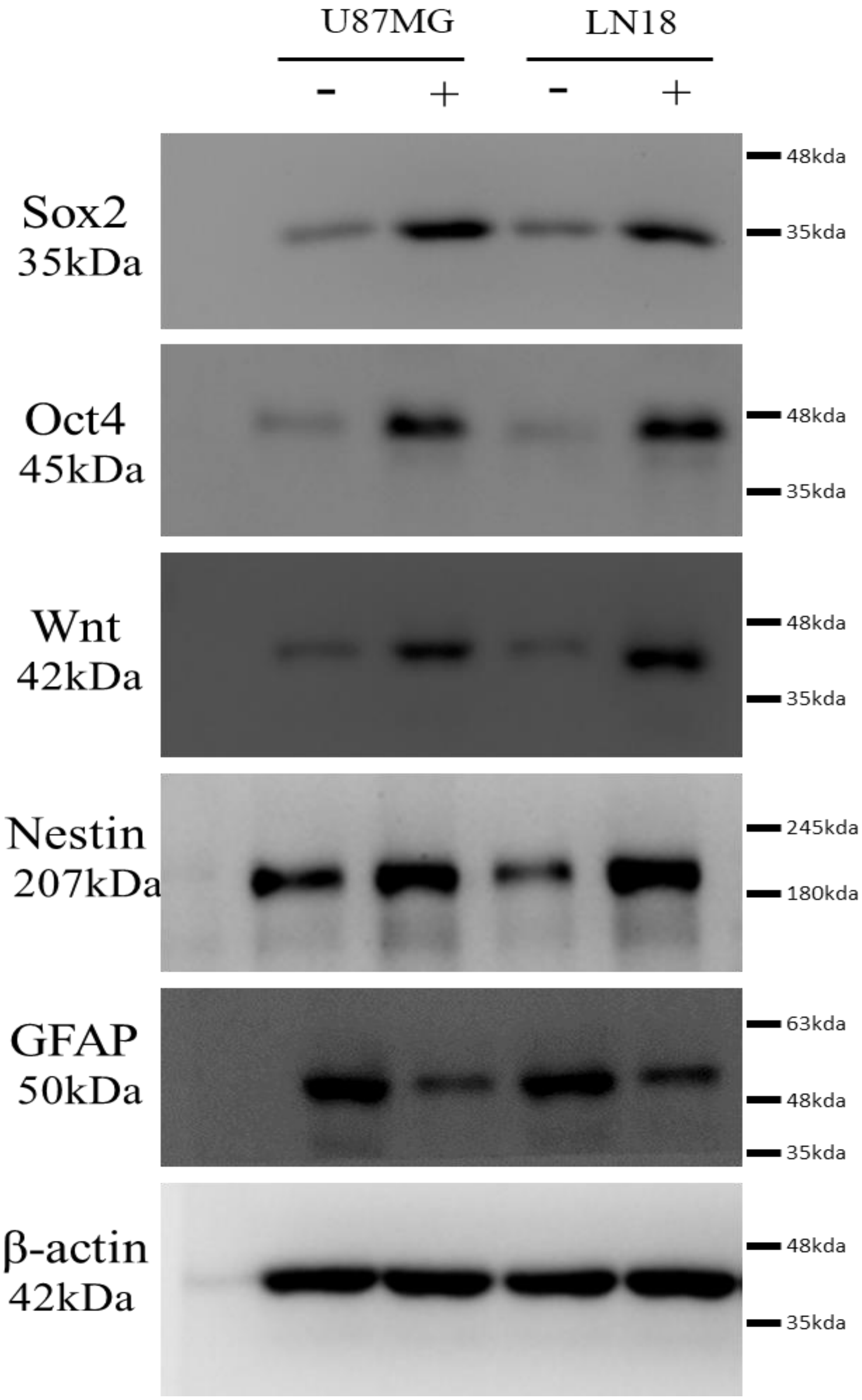

Supplementary Figure S1. Full-size blots of Figure 1D

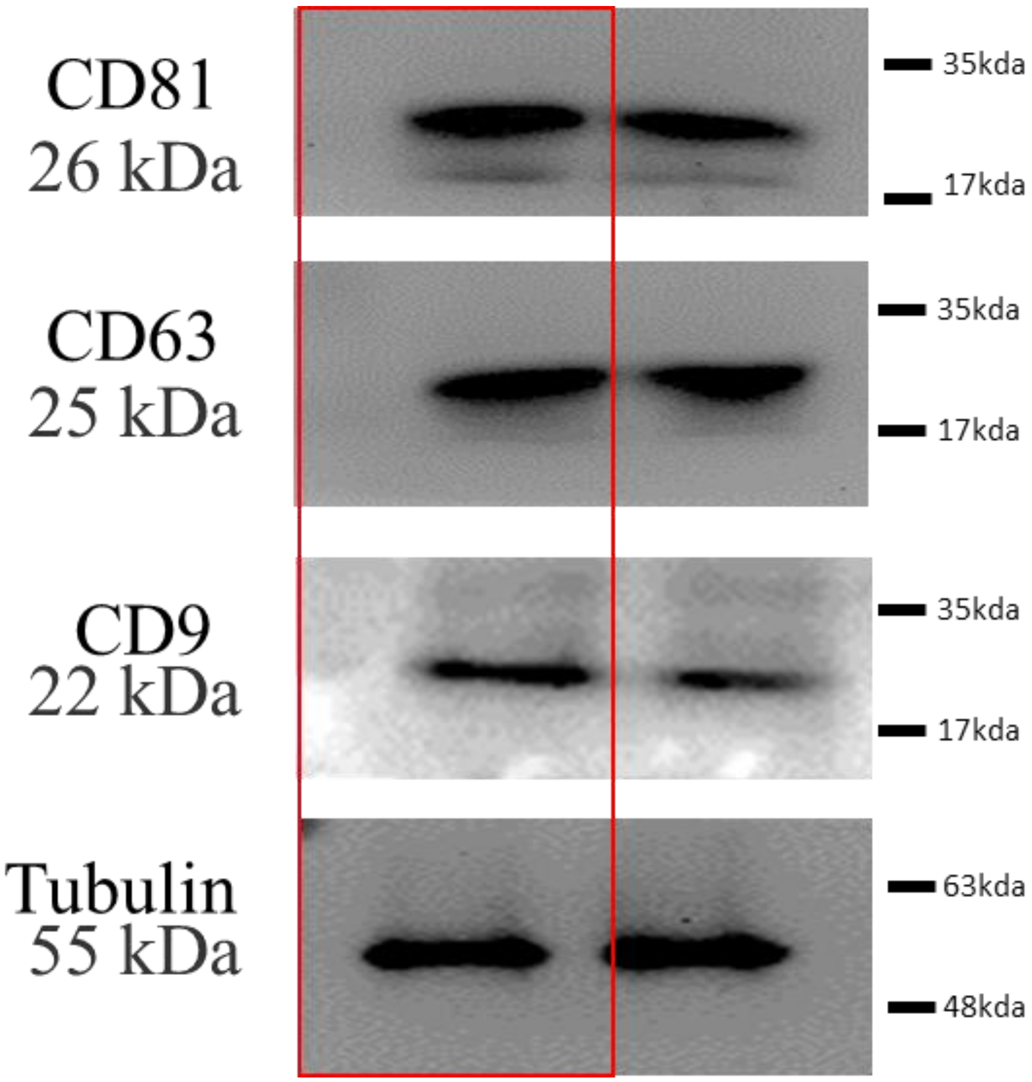

Supplementary Figure S2. Full-size blots of Figure 2A

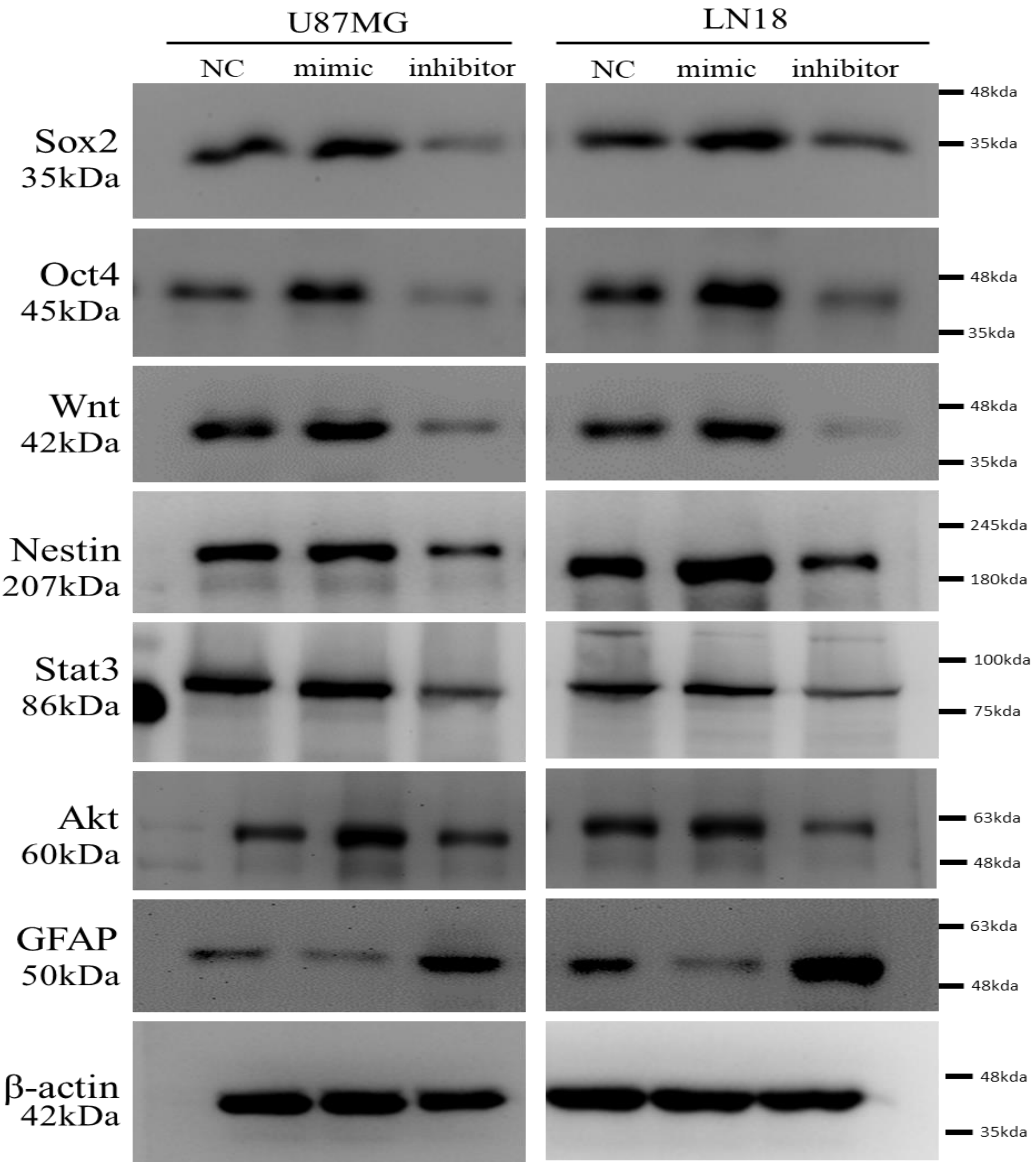

Supplementary Figure S3. Full-size blots of Figure 3C

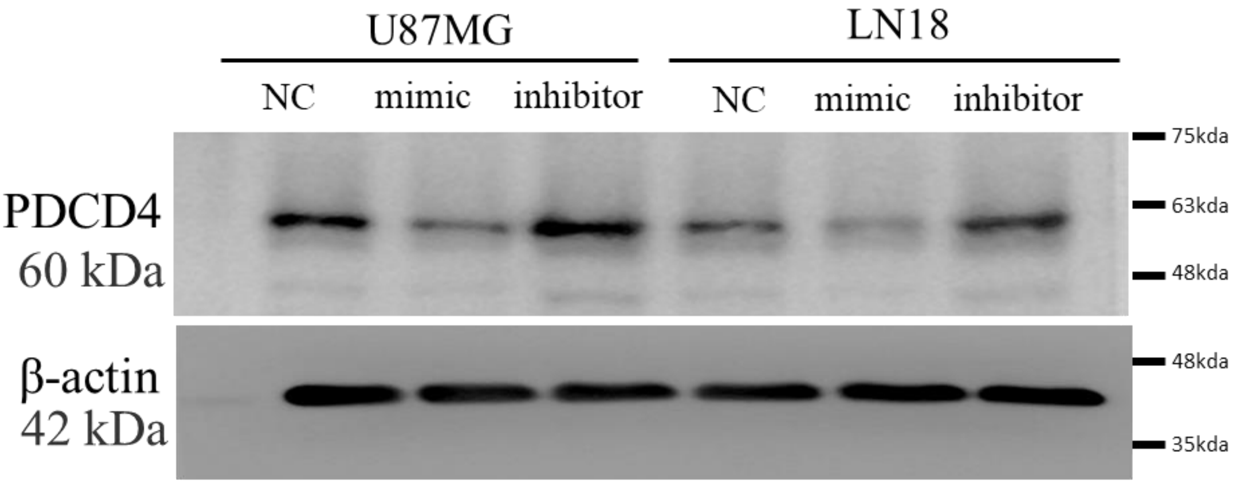

Supplementary Figure S4. Full-size blots of Figure 4C

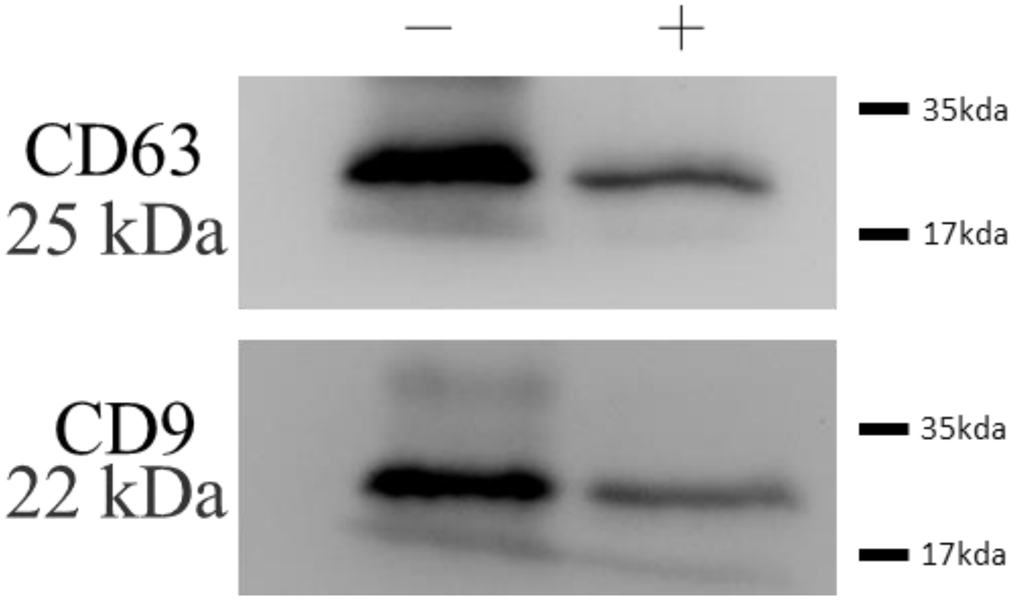

Supplementary Figure S5. Full-size blots of Figure 5F

Transfer the tumor to a culture dish
↓
Cut into small pieces
↓
Transfer the tumor pieces to a 15 ml tube
↓
Red blood cell lysis
↓
Enzymatic disaggregation (Trypsin and collagenase)
↓
FACS Staining and Cell Sorting
↓
GAMs are defined as CD11b$^+$ CD14$^+$ CD163 $^+$ CX3CR1 $^+$ HLA-DR $^+$
↓
GAMs

Transfer the tumor to a culture dish
↓
Cut into small pieces
↓
Transfer the tumor pieces to a 15 ml tube
↓
Red blood cell lysis
↓
Enzymatic disaggregation (Trypsin and collagenase)
↓
Incubate
↓
Primary culture

Supplementary Figure S6. Flow chart of GBM cell line and GAMs from clinical human GBM specimen isolation.

GAMs cultured in serum-free medium for 48h

↓

Collected GAMs cultured medium supernatant

↓

Serial centrifugation (500 g for 10 min, 1200 g for 20 min and 10,000 g for 30 min)

↓

Filtration with 0.22 μm pore syringe

↓

centrifugation (100,000 g for 60 min)

↓

Washed in PBS three time

↓

centrifugation (100,000 g for 60 min)

↓

Exosome

Supplementary Figure S7. Flow chart of Exosome isolation

GBM cells were seeded and allowed to attach

↓

GBM cells were pre-treatment with GAMs-derived exosomes (20 μg/mL) for 48 h

↓

GBM cells were treatment with various concentration of TMZ

↓

Harvest for analysis

GBM cells were seeded and allowed to attach

↓

GBM cells were transfected with miR-21-5p mimic or inhibitor by Lipofectamine 3000 for 24h

↓

Harvest for experiment

Supplementary Figure S8. Flow chart of GBM cell lines either treated with exosomes or mimics or inhibitors

Supplementary Table S1. Primer sequences used in this study

Gene	Forward sequence	Reverse sequence
Sox2	GCCGAGTGGAAACTTTTGTCG	GGCAGCGTGTACTTATCCTTCT
STAT3	CAGCAGCTTGACACACGGTA	AAACACCAAAGTGGCATGTGA
IL-6	ACTCACCTCTTCAGAACGAATTG	CCATCTTTGGAAGGTTCAGGTTG
GAPDH	GAGTCAACGGATTTGTCGT	GACAAGCTTCCCGTTCTCAG
Nestin	CTGCTACCCTTGAGACACCTG	GGGCTCTGATCTCTGCATCTAC
PDCD4	GGGAGTGACGCCCTTAGAAG	ACCTTTCTTTGGTAGTCCCCTT
CD206	TCCGGGTGCTGTTCTCCTA	CCAGTCTGTTTTTGATGGCACT
TNF-α	GAGGCCAAGCCCTGGTATG	CGGGCCGATTGATCTCAGC

Supplementary Table S2. Western blot antibodies sheet

No.	Target	Dilution	Source	
1	Sox2	1:1000	ab137385	Abcam
2	Oct4	1:1000	ab18976	Abcam
3	Wnt	1:1000	ab228526	Abcam
4	Nestin	1:1000	ab105389	Abcam
5	GFAP	1:1000	ab33922	Abcam
6	CD81	1:500	10630D	Thermo
7	CD63	1:500	10628D	Thermo
8	CD9	1:500	AHS0902	Thermo
9	PDCD4	1:1000	#9535	cellsignaling
10	Stat3	1:1000	#9132	cellsignaling
11	Tubulin	1:10000	11224-1-AP	PROTEINTECH
12	β-actin	1:10000	66009-1-Ig	PROTEINTECH

.
